# Vaccination of Macaques with DNA Followed by Adenoviral Vectors Encoding Simian Immunodeficiency Virus (SIV) Gag Alone Delays Infection by Repeated Mucosal Challenge with SIV

**DOI:** 10.1128/JVI.00606-19

**Published:** 2019-10-15

**Authors:** Neil Almond, Neil Berry, Richard Stebbings, Mark Preston, Claire Ham, Mark Page, Debbie Ferguson, Nicola Rose, Bo Li, Edward T. Mee, Mark Hassall, Christiane Stahl-Hennig, Takis Athanasopoulos, Timos Papagatsias, Shanthi Herath, Adel Benlahrech, George Dickson, Andrea Meiser, Steven Patterson

**Affiliations:** a Division of Infectious Disease Diagnostics, National Institute for Biological Standards and Controls, Potters Bar, Hertfordshire, United Kingdom; b Biotherapeutics Group, National Institute for Biological Standards and Controls, Potters Bar, Hertfordshire, United Kingdom; c Analytical Sciences Group, National Institute for Biological Standards and Controls, Potters Bar, Hertfordshire, United Kingdom; d Division of Virology, National Institute for Biological Standards and Controls, Potters Bar, Hertfordshire, United Kingdom; e German Primate Centre, Gottingen, Germany; f School of Biological Sciences, Royal Holloway, University of London, Surrey, United Kingdom; g Centre for Immunology and Vaccinology, Imperial College London, London, United Kingdom; Emory University

**Keywords:** vaccine, SIV, gag, DNA vaccine, adenovirus vector, simian immunodeficiency virus

## Abstract

Vaccines aimed at inducing T cell responses to protect against human immunodeficiency virus (HIV) infection have been under development for more than 15 years. Replication-defective adenovirus (rAd) vaccine vectors are at the forefront of this work and have been tested extensively in the simian immunodeficiency virus (SIV) challenge macaque model. Vaccination with rAd vectors coding for SIV Gag or other nonenvelope proteins induces T cell responses that control virus load but disappointingly is unsuccessful so far in preventing infection, and attention has turned to inducing antibodies to the envelope. However, here we report that Mauritian cynomolgus macaques (MCM), Macaca fascicularis, vaccinated with unmodified SIV *gag* alone in a DNA prime followed by an rAd boost exhibit increased protection from infection by repeated intrarectal challenge with low-dose SIVmac251. There was no evidence of infection followed by eradication. A significant correlation was observed between cytokine expression by CD4 T cells and delayed infection. Vaccination with *gag* fused to the ubiquitin gene or fragmented, designed to increase CD8 magnitude and breadth, did not confer resistance to challenge or enhance immunity. On infection, a significant reduction in peak virus load was observed in all vaccinated animals, including those vaccinated with modified *gag*. These findings suggest that a nonpersistent viral vector vaccine coding for internal virus proteins may be able to protect against HIV type 1 (HIV-1) infection. The mechanisms are probably distinct from those of antibody-mediated virus neutralization or cytotoxic CD8 cell killing of virus-infected cells and may be mediated in part by CD4 T cells.

**IMPORTANCE** The simian immunodeficiency virus (SIV) macaque model represents the best animal model for testing new human immunodeficiency virus type 1 (HIV-1) vaccines. Previous studies employing replication-defective adenovirus (rAd) vectors that transiently express SIV internal proteins induced T cell responses that controlled virus load but did not protect against virus challenge. However, we show for the first time that SIV *gag* delivered in a DNA prime followed by a boost with an rAd vector confers resistance to SIV intrarectal challenge. Other partially successful SIV/HIV-1 protective vaccines induce antibody to the envelope and neutralize the virus or mediate antibody-dependent cytotoxicity. Induction of CD8 T cells which do not prevent initial infection but eradicate infected cells before infection becomes established has also shown some success. In contrast, the vaccine described here mediates resistance by a different mechanism from that described above, which may reflect CD4 T cell activity. This could indicate an alternative approach for HIV-1 vaccine development.

## Abstract (precis)

The simian immunodeficiency virus (SIV) macaque model represents the best animal model for testing new human immunodeficiency virus type 1 (HIV-1) vaccines. Previous studies employing replication-defective adenovirus (rAd) vectors that transiently express SIV internal proteins induced T cell responses that controlled virus load but did not protect against virus challenge. However, we show for the first time that SIV *gag* delivered in a DNA prime followed by a boost with an rAd vector confers resistance to SIV intrarectal challenge. Other partially successful SIV/HIV-1 protective vaccines induce antibody to the envelope and neutralize the virus or mediate antibody-dependent cytotoxicity. Induction of CD8 T cells which do not prevent initial infection but eradicate infected cells before infection becomes established has also shown some success. In contrast, the vaccine described here mediates resistance by a different mechanism from that described above, which may reflect CD4 T cell activity. This could indicate an alternative approach for HIV-1 vaccine development.

## INTRODUCTION

Early studies of human immunodeficiency virus (HIV) infection provided good evidence that virus-specific CD8 cells are associated with control of virus load in infected individuals (1, 2), and consequently this focused attention on the development of vaccines to stimulate T cell CD8 responses (3). The macaque simian immunodeficiency virus (SIV) challenge model, initially involving intravenous challenge but now largely replaced by repeated low-dose mucosal challenge (4, 5), has been widely used for HIV vaccine development and testing. In this vaccine model, T cell control of virus load has been achieved by vaccination with replication-defective adenovirus (rAd) vectors and other nonpersistent virus vectors carrying genes for internal virus proteins; however, animals have not been protected against infection (3, 5–7). Resistance to virus challenge has been observed only when the vaccine included the envelope that induces neutralizing antibodies (8–12). Despite this, the potential of vaccine-induced CD8 cells to prevent the establishment of a persistent infection is highlighted by the work of Picker’s group, where a simian cytomegalovirus vaccine vector expressing SIV genes induced SIV-specific CD8 cells which protected 50% of vaccinated animals (13–16). Unlike vaccines that induced neutralizing antibodies, this vaccine did not prevent initial infection but eradicated the virus, likely by killing virus-infected cells before chronic infection was established. Cytomegalovirus (CMV) causes lifelong infection that probably results in lifetime expression of vaccine genes, and this may, in addition to unusual CD8 responses that recognize peptides associated with HLA-E and major histocompatibility complex (MHC) class II, explain the success of the vaccine. However, there are concerns about vaccinating with a vector that causes lifelong infection and difficulty in inducing HLA-E and MHC class II CD8 T cell-restricted responses in humans (17).

A problem with interpreting the results of vaccination experiments in small numbers of outbred rhesus macaques is that data may be skewed by differences in the range of MHC haplotypes present in different experimental groups. This difficulty can be alleviated by the use of Mauritian cynomolgus macaques (MCM), which are descended from a small number of animals introduced into Mauritius over the last 500 years and comprised of only seven main MHC haplotypes (18, 19). This has enabled us to design experiments employing several different haplotypes which are represented equally in different experimental groups. By applying this approach, we found evidence that vaccination with vectors carrying the *gag* gene alone can delay infection from low-dose mucosal SIV challenge and reduce peak virus load. Furthermore, the mechanism of protection from infection may be distinct from that mediated by antibody or the CD8 T cell killing of virus-infected cells.

## RESULTS

Three vaccines were tested, full-length SIVmac239 *gag* (group A), full-length SIVmac239 *gag* fused to the ubiquitin gene at the N terminus (group B), and 7 *gag* mini gene fragments spanning the whole of the *gag* gene with each fused to the ubiquitin gene at the N terminus (group C). These ubiquitin gene fusions were designed to enhance the magnitude of the CD8 response by promoting targeting of antigens to the proteasome and MHC class I processing pathway (20–22). The gene fragmentation strategy aimed to increase the breadth of the response by reducing the number of *gag* epitopes expressed by individual antigen-presenting cells (APC), thereby decreasing competition between different T cell clones (23–26). Vaccine delivery was by intradermal (i.d.) injection in order to target a greater number of dendritic cells.

### Vaccination with unmodified *gag* delays infection from intrarectal SIV challenge.

**FIG 1:**
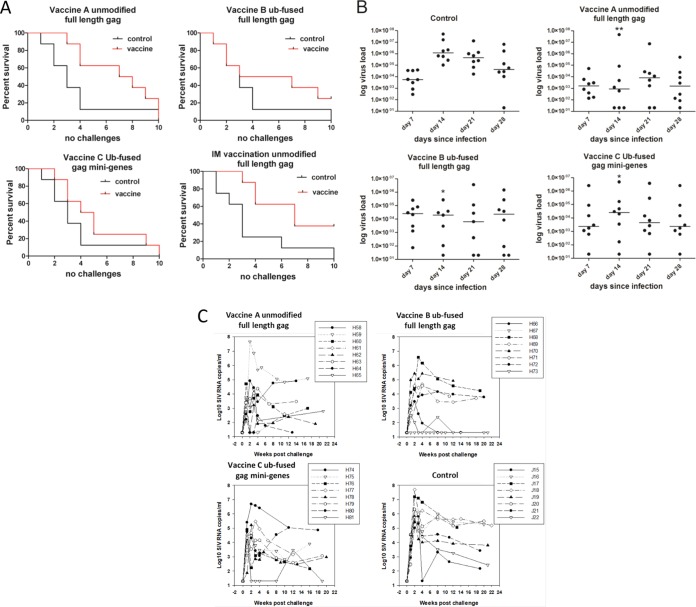
*gag* vaccination delays infection from intrarectal challenge with low-dose SIV and reduces virus load. (A) Kaplan-Meier survival curves showing time to infection, indicated as the number of weekly challenges, for vaccinated animals (red lines) and controls (black lines). Animals vaccinated i.d. with full-length unmodified *gag* gene resisted infection (*P* = 0.0081, which remained significant after Bonferroni’s correction). Animals vaccinated i.d. with the full-length *gag* gene fused at the N terminus to the ubiquitin (Ub) gene (*P* = 0.313) and animals vaccinated i.d. with 7 mini genes spanning the whole of *gag* with each gene fused to the ubiquitin gene at the N terminus (*P* = 0.139) were not protected. Animals vaccinated i.m. with full-length unmodified *gag* gene resisted infection (*P* = 0.023). The 16 controls used for these analyses combined the 8 naive controls challenged in parallel with vaccine groups A to C and a separate group of 8 naive controls challenged in parallel with the IM vaccinations (group D), which were performed subsequently to vaccinations A to C. Significance between curves was determined by the Gehan-Breslow-Wilcoxon test. (B) *gag* vaccination suppresses peak plasma virus RNA load. Median and individual virus loads are shown on days 7, 14, 21, and 28 for control and vaccinated animals and peaks at day 14 in controls. In all three vaccine groups, the virus load is significantly lower at day 14. Animals were vaccinated i.d. with full-length unmodified *gag* gene (*P* < 0.01), full-length *gag* gene fused at the N terminus to the ubiquitin gene (*P* < 0.05), and 7 mini genes spanning the whole of *gag* with each gene fused to the ubiquitin gene at the N terminus (*P* < 0.01). Results were analyzed by the Kruskal-Wallis test with Dunn’s multiple comparison correction. *, *P* < 0.05; **, *P* < 0.01. (C) Virus load for each macaque over time infected. Individual plasma viral RNA kinetics for vaccine groups A to C are compared to those of naive unvaccinated control animals. For each macaque, data are plotted from the time of the first positive qRT-PCR and sequentially thereafter, showing the time course of infection kinetics for each animal, in most cases up to 20 weeks postinfection.

Repeated intrarectal low-dose challenge with SIV resulted in 7 of 8 control unvaccinated animals becoming infected after 4 weekly challenges, with the remaining individual becoming infected in the 10-week challenge. In the animals vaccinated with the full-length unmodified *gag* (group A), only 3 of 8 were infected after the 4th challenge (Fig. 1A). Although all animals in this vaccinated group eventually became infected, they showed resistance to virus challenge. However, animals vaccinated with ubiquitin gene and mini gene *gag* constructs, groups B and C, designed to improve immune responses, showed only marginally higher levels of resistance than the unvaccinated controls, which were not statistically significant. Since vaccines were delivered i.d., we wondered whether the observed protection was associated with the route of vaccination. To test this hypothesis, unmodified full-length *gag* vaccine was delivered intramuscularly (i.m.) using the same vaccination regime (group D). Upon challenge of a new group of controls, 6 of 8 controls became infected by the third challenge and all 8 were infected by the 10th challenge, whereas 3 of 8 vaccinated animals remained uninfected after 10 challenges. By combining the outcomes of challenge with the full-length *gag* vaccines by the i.d. and i.m. routes with the outcomes of the 16 challenge controls in these studies, significant protection was observed with this vaccine (*P* = 0.0081 and *P* = 0.023, for i.d. and i.m. vaccinated animals, respectively).

### Vaccination reduces virus load in acute infection.

Early time (days 7 to 28) viral RNA (vRNA) profiles for vaccine groups (A to C) and naive challenge controls following infection are shown in Fig. 1B. Analyses of virus loads in individual animals up to week 22 are presented Fig. 1C. Virus loads in control and all vaccinated groups were not statistically significantly different on day 7 postinfection, nor between male and female vaccinated animals. In the unvaccinated control group, there was a marked increase in virus load from a median of 5.6 × 10^3^ SIV RNA copies/ml on day 7 to 1.2 × 10^6^ SIV RNA copies/ml on day 14, which was not mirrored by the vaccinated groups (Fig. 1B). All vaccine groups had statistically significant lower levels of virus than the control group on day 14 (A, *P* < 0.01; B and C, *P* < 0.05). At this time, the median virus loads were as follows: group A, 3 × 10^3^ SIV RNA copies/ml; group B, 2.9 × 10^4^ SIV RNA copies/ml; and group C, 2.5 × 10^4^ SIV RNA copies/ml. At day 28, the virus load in the control group (median, 4.2 × 10^4^ SIV RNA copies/ml) remained higher than the levels in the vaccinated group A (median, 1.3 × 10^3^ SIV RNA copies/ml), group B (median, 2.3 × 10^4^ SIV RNA copies/ml), and group C (median, 2.3 × 10^3^ SIV RNA copies/ml). Thus, vaccination with all *gag* constructs resulted in more effective control of the SIV virus load. Nevertheless, vaccination with the modified *gag* constructs did not confer superior control of virus load compared with individuals vaccinated with unmodified *gag*; indeed, the converse was more apparent.

### Vaccine-induced changes in *gag* coding sequences.

As vaccination blunted the rise in plasma virus load, we speculated that selective pressure was exerted on virus *gag*. Thus, full-length *gag* sequences were recovered as a single amplicon by reverse transcription PCR (RT-PCR) when the virus load was at or close to its peak and subjected to Illumina-based sequencing. SIVmac251 database reference sequences were used initially to align sequences recovered from the challenge virus stock. Consensus data based on sequences recovered from the challenge virus (SIVmac251; CSH stock) were aligned with the SIVmac251 reference (GenBank accession no. M19499), and the frequencies of single-nucleotide polymorphisms (SNPs) present in unvaccinated control macaques and vaccinated animals were assessed.

**FIG 2:**
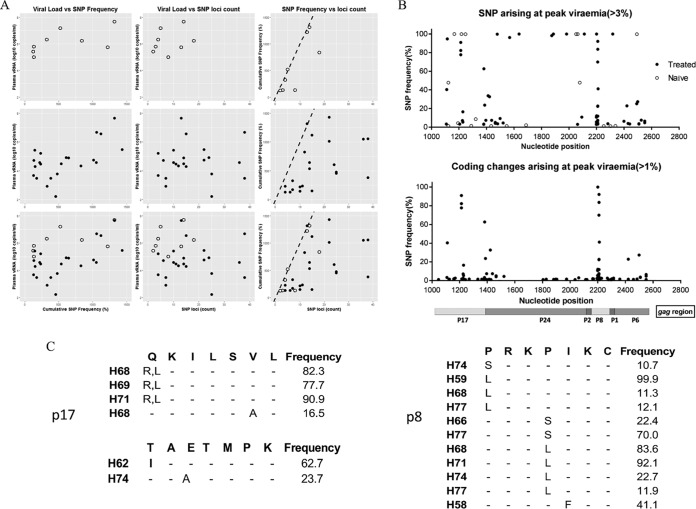
Nucleotide and amino acid changes across *gag* (Gag) in infected vaccinated and control animals. (A) Relationships between SNP frequency and viral load SNP loci across *gag* determined with a variant frequency greater than 1% were counted and summed across loci. These were plotted to the viral load of the sample and to each other represented as (i) vRNA load against SNP frequency, (ii) viral load against SNP loci count, and (iii) SNP frequency versus loci count; dotted lines indicate the position at which all loci were fixed at 100%. (B) Upper plot, SNP changes across *gag*. The frequency and position of SNPs not present in the challenge inocula are shown. Samples are shown against their frequency above 3% for vaccine-treated (closed circles) and naive (open circles) animals. Lower plot, positions of nonsynonymous changes and their frequency above 1% in treated vaccinated animals with an identifiable amino acid change. A schematic of the regions of the *gag* polyprotein are shown. (C) Identified high-frequency mutations in p17 and p6 regions in *gag*-vaccinated animals are shown. Three short regions corresponding to *gag* where samples have nonsynonymous changes at frequencies above 10% and not seen in the inocula are shown for matrix p17 (amino acids 58 to 65 and 114 to 121) and in nucleocapsid p8 (amino acids 387 to 394).

Figure 2A shows three separate analyses for naive-alone, vaccine-alone, or combined naive and vaccine groups and compares (i) vRNA levels with cumulative SNP percentages, (ii) vRNA levels with number of SNP loci identified, and (iii) cumulative SNP percentages with the number of SNP loci. A weak relationship between viral load and either cumulative SNP percentage or number of SNP loci is indicated (Fig. 2A), most likely reflecting the consistently high vRNA values obtained. However, a strong relationship between cumulative SNP percentage and the number of SNP loci was apparent.

In the vaccine-alone group (Fig. 2A, middle panels), where there is a broader range of vRNA values, a stronger correlation between plasma vRNA load and SNP percentage or number of SNP loci was identified. Hence, as viral load increases during the most pronounced cases of vaccine breakthrough, the cumulative percentage of SNPs identified increases proportionately, indicating a direct relationship between virus load and SNP generation.

### Coding changes arising at or around peak viremia.

Figure 2B, top panel, depicts the frequency of SNPs at or around the peak of viremia. In control and vaccinated macaques, SNPs at a frequency equal to or greater than 3% that are not present in the challenge inoculum are typically not observed in the first vRNA detected in the infected animal. Coding changes present at a frequency of 1% or greater that are not present in the challenge inoculum are shown in Fig. 2B, lower panel. In the latter analysis, taking an arbitrary cutoff of SNP frequencies of 10% or greater coding for predicted amino acid changes, three key regions were identified. Interestingly, none of these were located in the p24 capsid region, but rather in p17 (matrix) and p8 (nucleocapsid). Figure 2C indicates specific motifs where repeated changes have been identified. Outliers in group A and group B (H59 and H68, respectively) identify with unique mutations.

### CD4 and CD8 T cell responses to vaccination.

**FIG 3:**
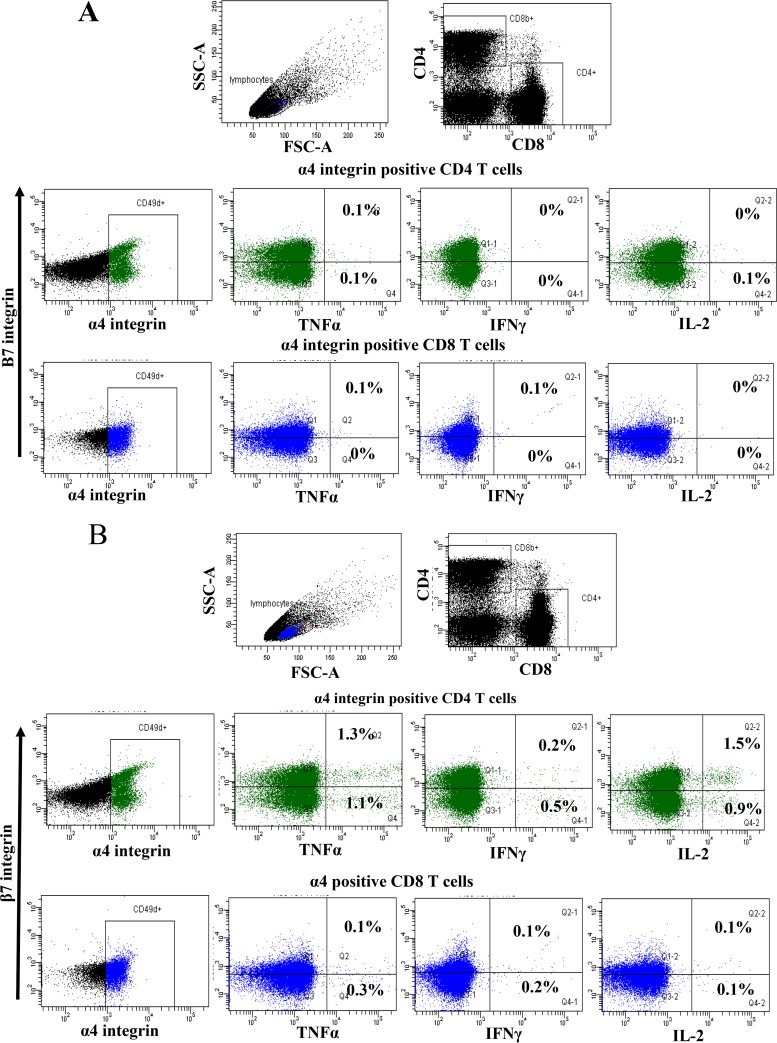
Flow cytometry analysis from a representative immunized macaque. (A) Plot of PBMCs from a vaccine group A animal (H58) at day 147 showing α4 integrin-positive CD4 and CD8 cells staining for TNF-α, IFN-γ, or IL-2 after stimulation with the DMSO control. (B) Flow cytometry plot of PBMCs from a vaccine group A animal (H58) at day 147 showing α4 integrin-positive CD4 and CD8 cells staining for TNF-α, IFN-γ, or IL-2 after stimulation with a p17 peptide pool.

**FIG 4:**
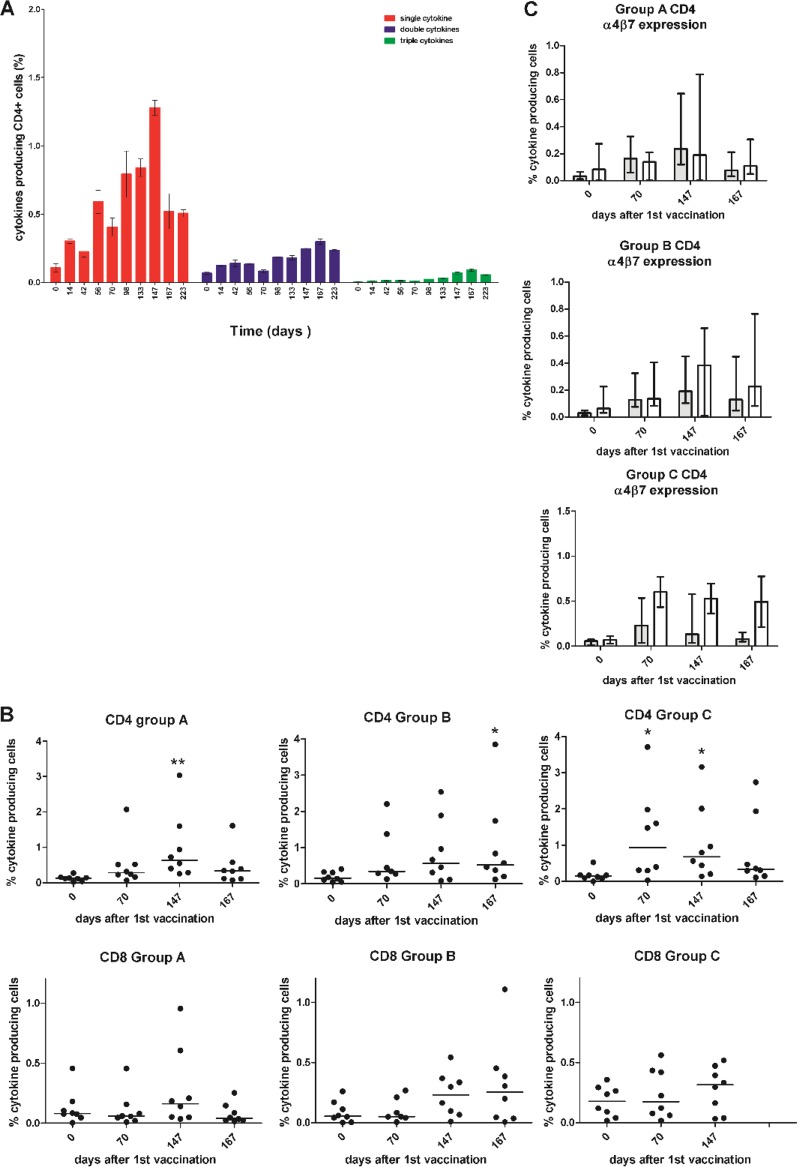
T cell cytokine analysis. (A) CD4 T cells producing 1, 2, or 3 cytokines shown by a representative vaccine group A animal (H58). Means and standard errors (SE) are shown for results from triplicate samples. (B) The percentage of cytokine-producing CD4 and CD8 T cells (IFN-γ, IL-2, or TNF-α) in vaccinated animals is shown for the three vaccine groups: animals vaccinated i.d. with the full-length unmodified *gag* gene, those vaccinated with the full-length *gag* gene fused at the N terminus to the ubiquitin gene, and those vaccinated with 7 mini genes spanning the whole of *gag* with each gene fused to the ubiquitin gene at the N terminus. *, *P* < 0.05; **, *P* < 0.01. Data were analyzed by the Kruskal-Wallis test with Dunn’s multiple comparison correction. Immunological analyses were not performed in animals from the i.m. vaccinated group D. (C) Percentage of cytokine-positive cells (median and interquartile range) that are α4β7 positive (filled bars) or negative (open bars) for CD4 T cells for vaccine groups A, B, and C.

Cryopreserved peripheral blood mononuclear cells (PBMCs) from vaccinated animals were stimulated with *gag* peptide pools and analyzed by flow cytometry to detect production of gamma interferon (IFN-γ), tumor necrosis factor alpha (TNF-α), or interleukin 2 (IL-2). Cytokine-producing cells were found to be mainly α4 integrin expressing (Fig. 3A, dimethyl sulfoxide [DMSO] control; Fig. 3B, peptide stimulated), and a higher percentage of cytokine-positive CD4 than CD8 T cells was observed. Across all vaccine groups, expression of TNF-α and IL-2 tended to be higher than that of IFN-γ in CD4 cells, whereas CD8 cells expressed similar levels of TNF-α and IFN-γ but lower numbers of cells expressed IL-2. A fraction of the stimulated cells produced two or three cytokines, as shown by CD4 T cells from a representative vaccine group A animal (H58) (Fig. 4A). Analysis of the number of cells producing any of the three cytokines is shown prevaccination (day 0), 14 days after the third DNA vaccination (day 70), 7 days after the adenovirus vector vaccine boost (day 147), and immediately prior to virus challenge (day 167) (Fig. 4B). The CD4 cells in all three vaccination groups showed an increase in the number of cytokine-producing cells after vaccination, which was significantly different from prevaccination levels for at least one time point after the third DNA prime, with one group showing significant cytokine production after the third DNA vaccination (group A at day 147, *P* < 0.01; group B at day 167, *P* < 0.05; group C at day 70, *P* < 0.05; and group C at day 147, *P* < 0.05). The measured CD8 responses were low, being barely above that observed prior to vaccination, and did not reach statistical significance from prevaccination levels for any group or time point. The modified *gag* vaccines did not induce an observable improvement in the immune response for either CD4 or CD8 T cells (Fig. 4B). Although the vaccine was not administered by a mucosal route, a high proportion of the vaccine-specific CD4 T cells expressed the mucosal homing marker α4β7 integrin. There was a trend for vaccine group A cytokine-producing CD4 T cells to show a higher proportion, usually more than 50%, of α4β7 integrin-expressing cells. This proportion was greater than that observed for vaccine groups B and C. In these groups, there was a greater proportion of α4β7-negative cells (Fig. 4C). CD8 cells bearing α4β7 always represented a minor population of antigen-specific cells for all vaccine groups (Fig. 3A and B, lower panels with blue scatter points).

**FIG 5:**
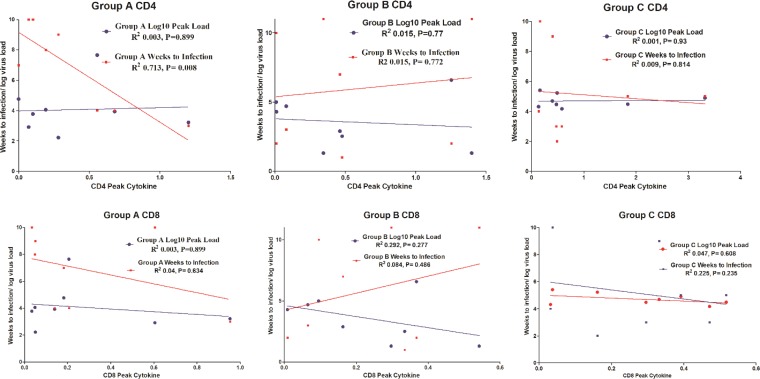
Correlation between peak cytokine response and time to infection/peak virus load. The highest percentage of CD4 and CD8 T cells secreting cytokine following vaccination (peak cytokine production) is plotted against time in weeks to infection (red lines) and against peak virus load (blue lines) for vaccine groups A, B, and C. For the CD4 response in vaccine group A, there is a significant correlation with time to infection after weekly challenges (*P* = 0.008, *r*^2^ = 0.7). There is no significant correlation of these parameters in vaccine groups B and C. There is no significant correlation between peak cytokine production and time to infection for CD8 T cells from any vaccine group. Similarly, there is no significant correlation between peak cytokine production and peak virus load for CD4 or CD8 cells from any vaccination group.

The relationships between peak total cytokine response and time to infection or peak virus load were investigated for all vaccine groups (Fig. 5). A significant correlation was found in vaccine group A, the only group exhibiting significant resistance against virus challenge, between peak cytokine response and time to infection for CD4 T cells (*P* = 0.0083, *R*^2^ = 0.7132) but not for CD8 T cells. No significant correlation was observed between time to infection or peak virus load and cytokine production for CD4 T cells or CD8 T cells in vaccine group B or C.

## DISCUSSION

Vaccination with *gag* delivered in a replication-defective adenovirus vector has previously been shown to reduce virus load in SIV-challenged rhesus macaques but not to protect against infection (3, 5, 27). Similarly, we found that all three of our test DNA prime-adenovirus boost vaccines reduced the SIV virus load in comparison to that of unvaccinated controls. However, in marked contrast with other adenovirus vector vaccination studies with nonenvelope genes, we observed delayed infection after repeated low-dose intrarectal challenge. In view of the resistance conferred by vaccine A to challenge with a homologous virus, it would be interesting to investigate a more demanding heterologous challenge. A previous study using a fowlpox/rAd combination to deliver *gag*, *pol*, and *env* genes reported a delay in acquisition despite neutralizing antibodies being low or absent (28). Surprisingly, protection was limited to the unmodified *gag* constructs, despite the fact that all vaccine constructs suppressed the virus load. In previous *in vitro* experiments, fusion of *gag* to the ubiquitin gene, either full length or fragmented, was found to increase proteasomal targeting as expected (23, 29), but *in vivo*, this did not correlate with improvement in the CD8 responses or the ability to control the virus load, compared with animals vaccinated with the unmodified *gag*. Interestingly, a failure to enhance the CD8 response has also been reported for fusion of ubiquitin to hepatitis C core protein (30). Improvement of T cell responses by fusion to ubiquitin has been shown *in vivo* for other antigens (20), and in an *in vitro* priming system fragmentation was observed to broaden the response (23). Whether fusion to ubiquitin enhances immunogenicity may depend on the protein to which it is fused. In support of this hypothesis, we found that the ubiquitin gene fused to *gag* but not the melanin A gene failed to enhance CD8 responses. We also observed that human dendritic cells transfected with the ubiquitin gene-fused *gag* failed to mature fully, compared with those transfected with unmodified *gag*, and in addition were observed to produce more IL-10. These findings may explain the current observations (29).

One advantage of the Mauritian cynomolgus macaque model is the limited repertoire of MHC haplotypes which may impact the ability to control SIV infection; hence, to mitigate any overt influence on outcomes, the haplotype frequency was relatively evenly distributed among study groups. Next-generation sequence analysis of the *gag* region of plasma virus recovered during acute infection suggested that some coding sequence changes were associated with vaccine escape, identified predominantly in p17 (matrix) and p6 (nucleocapsid) at different frequencies in viral variants not present in the inoculum in breakthrough cases. Several coding changes were mapped immediately upstream of the first zinc finger motif in the SIV NC, for example. Overall, an increased plasma virus load was linked with an increased cumulative percentage of SNPs, with a direct correlation between these two variables during the acute infection period as breakthrough virus was identified. We found no direct correlation between MHC haplotype and breakthrough frequencies, and interestingly the SNP calling rate was very low in p27 CA, with few novel variants identified across this region, where MHC-restricted T cell epitopes have been identified (31). Perhaps more notable are changes in the p17 matrix which map to at least one immunogenic region previously identified to accumulate a high frequency of mutations during acute SIV infection in MCM expressing an M3 haplotype (32). H68, with the highest acute virus load in group B, accumulated mutations in both Q58R and V63A during the first 3 weeks of infection. The significance of mutations in regions of zinc finger motifs that may lead to more efficient *gag* processing (33) would reflect our observations but remain unconfirmed. Moreover, if early CD8 T cell escape was a significant feature of vaccine breakthrough, a higher frequency of SNP variants in p27 CA might have been anticipated if under selective pressure during acute infection. The absence of immune pressure on p27 capsid but presence in other regions of *gag* suggest alternative mechanisms contributing to viral breakthrough, including CD4^+^ T cell responses which may contribute to viral control (29).

Unfortunately, the measured T cell responses in all the three vaccine groups were lower than expected and could be detected only by intracellular staining and flow cytometry using brefeldin A to prevent cytokine secretion. No significant responses were detected by enzyme-linked immunosorbent spot (ELISPOT) assay (data not shown), in which only a fraction of the secreted cytokine is detected and thus may be less sensitive than flow cytometry. Our findings may reflect the use of cryopreserved PBMCs, since a study reported after the completion of our vaccine challenge experiments (34) found that freezing lymphocytes from SIV-vaccinated macaques significantly reduced the breadth and magnitude of the immune response detected compared with that of fresh cells.

Protection mediated by neutralizing anti-envelope antibody is considered to block cellular entry, thus preventing initiation of productive infection. In contrast, the only SIV T cell vaccine to date that confers protection from challenge, the CMV vector vaccine, does not prevent initial infection but rather eradicates infected cells (13–15). In the present study, there was no envelope in the vaccine, thus ruling out protection by neutralizing antibody, and there was no evidence of infection followed by eradication, suggesting that CD8 cells did not mediate protection. We speculate that resistance to virus challenge may reflect production of T cell-derived CCR5-blocking chemokines such as RANTES (35) and may result in protection from a broader spectrum of virus strains. This idea is supported by findings from an intravaginal HIV vaccine study which found increased secretion of CCR5 binding CC chemokines that correlated with reduced HIV infection *in vitro* (36). Alternatively, an increased antiviral state in CD4^+^ cells which reduced their capacity to initiate productive infection following intrarectal challenge may have been established. It is relevant that in vaccine group A, the only group in which there was delayed infection, there was a correlation between CD4 cytokine production and the number of SIV exposures required for infection. It is important to understand why the ubiquitin-modified vaccines failed to protect against challenge. Classically, MHC class II presentation is associated with exogenous antigen, but there is evidence of significant presentation of peptides derived from cytoplasmic antigen which may be mediated by autophagy (37). Thus, by modifying *gag* to target the proteasome, we may have altered to some extent the supply of intracellular antigen destined for presentation on MHC class II. This could explain the differences in protection mediated by our different vaccines and support the hypothesis that protection is mediated by CD4 T cells. Also of note was that most of the antigen-specific CD4 T cells in vaccine group A expressed the mucosa-homing receptors α4β7, whereas in vaccine groups B and C, they represented less than 50% of the antigen-specific cells. However, expression of α4β7 is associated with higher levels of CCR5 and increased susceptibility to HIV-1 infection (28). Thus, analysis of CCR5 expression in future work is of interest. Whether CD8 cells played a role in delaying infection through a noncytolytic mechanism such as that previously described (38) cannot be fully ruled out. However, we expect them to function in controlling virus load.

Delayed infection is unlikely to reflect an innate response induced by the adenovirus vector itself, since all vaccinated groups were vaccinated with rAd vectors. Intradermal vaccination, which aimed to target a greater number of dendritic cells, also did not appear to be a critical factor, since delayed infection was also observed in animals after i.m. vaccination with full-length unmodified *gag*. Future studies analyzing cytokine production after i.m. vaccination may prove informative.

One explanation for the different outcome from previous studies may reflect the host species used. The current study was performed in the Mauritian cynomolgus macaque model rather than in Indian rhesus macaques. A previous comparative study of SIVmac251 infection and control of viremia in rhesus and cynomolgus macaques identified an association between more effective control of viremia and a more vigorous early IFN-γ ELISPOT response, including to SIN Gag peptides (39). Moreover, the virus stock used was initially prepared on rhesus PBMCs; hence, the possibility exists that it may not be as well adapted for replication in MCM, which could account for apparently enhanced protection from the *gag*-only vaccine in this species. However, the SIVmac251 (CSH stock) challenge in naive MCM demonstrated a robust infection kinetics profile with a high peak viremia and persisting steady-state kinetics, demonstrating that this virus stock represents a robust challenge to evaluate and break down (40) protection in this species. Furthermore, attempts to increase the pathogenicity of SIV stocks by passage through cynomolgus macaques have had only a modest effect (40). Nevertheless, in view of this concern and the resistance conferred by vaccine A to challenge with this homologous virus, it would be interesting to investigate a more demanding heterologous challenge to further tease out potential correlates of protection.

This is the first report that macaques vaccinated with a DNA prime-rAd booster vaccination regimen carrying the *gag* gene alone can delay infection by mucosal challenge of SIV. The underlying protective mechanism is probably distinct from antibody-mediated virus neutralization or CD8 T cell killing of virus-infected cells and may be mediated by CD4 T cells, but elucidation of the precise details requires further study. However, it does keep open the possibility that vaccination with a nonpersistent virus vector expressing *gag* may be an important component of a future protective vaccine for HIV.

## MATERIALS AND METHODS

### Animals.

Male juvenile MCM used in this study were housed and maintained in accordance with UK Home Office guidelines for the care and maintenance of nonhuman primates. Vaccinated MCM were MHC haplotyped (M1 to M8) using previously reported microsatellite DNA techniques to reduce allele bias in the study design (41), and the haplotypes were evenly distributed across vaccine groups (Table 1). Males and females were also distributed evenly among vaccine groups. Blood samples were taken before and during the vaccination period and frozen for subsequent analysis.

**TABLE 1:** MHC haplotypes and sex distribution of animals in vaccine groups A, B, and C

Vaccine group and animal	MHC haplotype^*a*^	Sex
A		
H58	1/1	M
H59	2/5	M
H60	3/5	M
H61	1/4	M
H62	1/1	F
H63	1/3	F
H64	1/3	F
H65	3/3	F
B		
H66	1/1	M
H67	3/3	M
H68	3/5	M
H69	5/4	M
H70	1/1	F
H71	1/3	F
H72	1/3	F
H73	3/4	F
C		
H74	1/1	M
H75	1/1	M
H76	1/3	M
H77	1/5	M
H78	1/3	F
H79	3/3	F
H80	1/4	F
H81	3/4	F

a Haplotypes 1 to 5 constitute experimental groups.

### Vaccination.

Three vaccines were tested in groups of 8 MCM, with each animal being vaccinated i.d. or i.m. with 100-μg DNA primes at weeks 0, 4, and 8 and an rAd5 boost (6 × 10^10^ particles) at week 16. Eight unvaccinated animals served as controls for each group challenged with the same virus stock. The i.m. vaccination experiments were performed subsequent to the i.d. vaccinations and employed an additional group of 8 control animals that were challenged in parallel. The vaccines were as follows: full-length SIVmac239 *gag*, full-length SIVmac239 *gag* fused to the ubiquitin gene at the N terminus, and 7 *gag* mini gene fragments that were between 70 and 93 nucleotides in length spanning the whole of the *gag* gene and overlapping, by 10 nucleotides, each fragment being fused to the ubiquitin gene at the N terminus. The vaccines were given by i.d. injection to increase targeting of dendritic cells and in a subsequent experiment by the i.m. route. The mini gene vaccinations were given at seven separate sites.

### Virus challenge.

From week 23, the animals were challenged intrarectally with 150 50% tissue culture infective doses (TCID_50_) of SIVmac251 at weekly intervals for up to 10 weeks or until they became infected, as determined by virus in the plasma. The challenge virus represented an early passage of this prototype propagated on primary rhesus monkey PBMCs. Details of this particular stock have been previously described (42, 43).

### Vaccine constructs.

SIVmac251 *gag* sequences were cloned into a replication-deficient E1- and E3-deleted adenovirus type 5 vector (Ad5). Recombinant Ad5 vectors were generated by insertion of mRNA sequence-optimized Ub(G76V)-fused hemagglutinin (HA)-tagged SIV *gag* genes (GeneArt, Germany) into Ad5 shuttle vectors (pShuttle2; Capital Biosciences, USA) and ligation with Ad5 vector backbones, followed by large-scale production in packaging cell lines and virus purification (Vector Biolabs, USA). For DNA vaccination, vaccine genes were cloned into pcDNA3.1 and bulk preparation was outsourced (Aldevron, USA).

### Plasma viral RNA quantification.

Quantitative determinations of vRNA levels in plasma were made using a previously described quantitative reverse transcription PCR (qRT-PCR) method targeting SIV *gag* (44) using the RNA Ultrasense one-step RT-PCR kit (ThermoFisher Scientific) on an Mx3000P genetic analyzer (Stratagene); quantitative data were calculated using Mx3000P software. The lower level of detection of the qRT-PCR assay is 50 SIV RNA copies ml^−1^ plasma.

### Deep sequencing of SIV *gag*.

Total RNA isolated from plasma around peak viremia (between day 7 and 21) was assayed using the SuperScript III one-step RT-PCR system with Platinum *Taq* DNA polymerase high fidelity (ThermoFisher Scientific). Full-length SIV *gag* was amplified as a single amplicon using forward (CCTGAGTACGGCTGAGTGAA) and reverse (TGGACCTAACTCTATTCCTGTTACA) primers at 400 nM concentrations, with a thermoprofile of 55°C for 30 min, 94°C for 2 min, followed by 35 cycles (94°C for 15 s, 60°C for 30 s, and 68°C for 2 min, with elongation at 68°C for 5 min). Amplicons verified by agarose gel electrophoresis were quantified using Qubit double-stranded DNA (dsDNA) high-sensitivity (HS) fluorimetry (ThermoFisher Scientific). Bar-coded libraries of amplicons were made using the Nextera XT library preparation kit, with equimolar amounts of each sample sequenced in 250-bp paired-end MiSeq V500 sequencing reactions (both Illumina). SIVmac251 RNA from two separate virus inoculum aliquots were also extracted, and SIV *gag* was amplified in duplicate as for the samples isolated from the experimental animals. Viral isolate and plasma-derived sequences described in this report are located in the NCBI/GenBank database under Bioproject ID PRJNA528275.

### Quality control and SNP calling.

Sequencing reads were quality trimmed with Trimmomatic (45), data aligned with BWA (46), and sorted with Picard, and deduplicating was performed with Picard and Pileup with SAMtools; both paired and unpaired reads were included in the assembly, with paired-read information used where available. Variant data were collated in Excel, and SNPs were filtered for strand bias (≤90%) and frequency (≥1%), restricted to *gag* only (nucleotide positions 1041 to 2561, numbered as for SIVmm251 [GenBank accession number M19499]). Mean coverage was 20,546 (SD = 7,883; range, 478 to 3,914).

### Flow cytometry.

Cryopreserved PBMCs were thawed in RPMI 1640–15% fetal calf serum (FCS) and stimulated by synthetic peptide pools covering p24 and p17 sequences (1 μg/ml/peptide, 15mers with an 11-amino-acid overlap; supplied by the Centre for AIDS Reagents at the National Institute for Biological Standards and Control, UK) with costimulatory antibodies CD28 (BioLegend, UK) at 1 μg/ml. In the negative control, cells were incubated with 0.1% DMSO, and positive-control cells were incubated with 1 μg/ml *Staphyolococcus enterotoxin* (SEB). A 10-μg/ml concentration of brefeldin A (Sigma, UK) was used as a secretion inhibitor. After overnight incubation at 37°C, cells were labeled with the following combinations of surface makers: anti-CD4 allophycocyanin-Cy7 (Biolegend, UK) and anti-CD8b phycoerythrin (PE) (BD Bioscience, UK), and anti-CD49d peridinin chlorophyll protein (PerCP)-Cy5.5 (Biolegend, UK) and anti-integrin beta7 fluorescein isothiocyanate (FITC) (Biolegend, UK). Labeled cells were washed with phosphate-buffered saline (PBS)-FCS (5%) with 0.1% sodium azide and then fixed for 20 min at room temperature in Caltag fixation reagent A (Invitrogen, UK). The cells then were incubated with Caltag fixation reagent B (Invitrogen, UK) and anti-TNF-α PE-Cy7 (Biolegend, UK), anti-IFN-γ Pacific Blue (Biolegend, UK), and anti-IL-2 allophycocyanin (Biolegend, UK) for 30 min at room temperature. Labeled cells were fixed with 0.5% paraformaldehyde and analyzed using a BD FACSCanto II cytometer equipped with three lasers capable of simultaneously measuring eight parameters. At least 100,000 lymphocyte events were collected and analyzed using FACSDiva for subset analysis.

### Statistical analysis.

Survival after weekly low-dose intrarectal virus challenge was plotted as a Kaplan-Meier survival curve, and significance between curves was determined by the Gehan-Breslow-Wilcoxon test. For statistical analysis of survival, the data for the controls from the i.d. and i.m. vaccinations, 16 animals, were combined. Grouped data are expressed as medians. Comparisons between groups were performed by the Kruskal-Wallis test, followed by Dunn’s multiple comparison posttest. Statistical analyses were performed using GraphPad Prism 5 (GraphPad Software, La Jolla, CA, USA).

## References

[1] Borrow P, Lewicki H, Wei XP, Horwitz MS, Peffer N, Meyers H, Nelson JA, Gairin JE, Hahn BH, Oldstone MBA, Shaw GM 1997 Antiviral pressure exerted by HIV-1-specific cytotoxic T lymphocytes (CTLs) during primary infection demonstrated by rapid selection of CTL escape virus. Nat Med 3:205–211. doi:10.1038/nm0297-205. 9018240

[2] Koup RA, Safrit JT, Cao YZ, Andrews CA, Mcleod G, Borkowsky W, Farthing C, Ho DD 1994 Temporal association of cellular immune-responses with the initial control of viremia in primary human immunodeficiency virus type 1 syndrome. J Virol 68:4650–4655. https://jvi.asm.org/content/68/7/4650. 8207839 10.1128/jvi.68.7.4650-4655.1994 PMC236393

[3] Shiver JW, Fu T-M, Chen L, Casimiro DR, Davies M-E, Evans RK, Zhang Z-Q, Simon AJ, Trigona WL, Dubey SA, Huang L, Harris VA, Long RS, Liang X, Handt L, Schleif WA, Zhu L, Freed DC, Persaud NV, Guan L, Punt KS, Tang A, Chen M, Wilson KA, Collins KB, Heidecker GJ, Fernandez VR, Perry HC, Joyce JG, Grimm KM, Cook JC, Keller PM, Kresock DS, Mach H, Troutman RD, Isopi LA, Williams DM, Xu Z, Bohannon KE, Volkin DB, Montefiori DC, Miura A, Krivulka GR, Lifton MA, Kuroda MJ, Schmitz JE, Letvin NL, Caulfield MJ, Bett AJ, Youil R, Kaslow DC, Emini EA 2002 Replication-incompetent adenoviral vaccine vector elicits effective anti-immunodeficiency-virus immunity. Nature 415:331–335. doi:10.1038/415331a. 11797011

[4] Keele BF, Li H, Learn GH, Hraber P, Giorgi EE, Grayson T, Sun CX, Chen YL, Yeh WW, Letvin NL, Mascola JR, Nabel GJ, Haynes BF, Bhattacharya T, Perelson AS, Korber BT, Hahn BH, Shaw GM 2009 Low-dose rectal inoculation of rhesus macaques by SIVsmE660 or SIVmac251 recapitulates human mucosal infection by HIV-1. J Exp Med 206:1117–1134. doi:10.1084/jem.20082831. 19414559 PMC2715022

[5] Liu JY, O’Brien KL, Lynch DM, Simmons NL, La Porte A, Riggs AM, Abbink P, Coffey RT, Grandpre LE, Seaman MS, Landucci G, Forthal DN, Montefiori DC, Carville A, Mansfield KG, Havenga MJ, Pau MG, Goudsmit J, Barouch DH 2009 Immune control of an SIV challenge by a T-cell-based vaccine in rhesus monkeys. Nature 457:87–91. doi:10.1038/nature07469. 18997770 PMC2614452

[6] Mudd PA, Martins MA, Ericsen AJ, Tully DC, Power KA, Bean AT, Piaskowski SM, Duan LJ, Seese A, Gladden AD, Weisgrau KL, Furlott JR, Kim YI, de Santana MGV, Rakasz E, Capuano S, Wilson NA, Bonaldo MC, Galler R, Allison DB, Piatak M, Haase AT, Lifson JD, Allen TM, Watkins DI 2012 Vaccine-induced CD8(+) T cells control AIDS virus replication. Nature 491:129–U133. doi:10.1038/nature11443. 23023123 PMC3883109

[7] Cervasi B, Carnathan DG, Sheehan KM, Micci L, Paiardini M, Kurupati R, Tuyishime S, Zhou XY, Else JG, Ratcliffe SJ, Ertl HC, Silvestri G 2013 Immunological and virological analyses of rhesus macaques immunized with chimpanzee adenoviruses expressing the simian immunodeficiency virus Gag/Tat fusion protein and challenged intrarectally with repeated low doses of SIVmac. J Virol 87:9420–9430. doi:10.1128/JVI.01456-13. 23804645 PMC3754116

[8] Barouch DH, Liu JY, Li HL, Maxfield LF, Abbink P, Lynch DM, Iampietro MJ, SanMiguel A, Seaman MS, Ferrari G, Forthal DN, Ourmanov I, Hirsch VM, Carville A, Mansfield KG, Stablein D, Pau MG, Schuitemaker H, Sadoff JC, Billings EA, Rao M, Robb ML, Kim JH, Marovich MA, Goudsmit J, Michael NL 2012 Vaccine protection against acquisition of neutralization-resistant SIV challenges in rhesus monkeys. Nature 482:89–93. doi:10.1038/nature10766. 22217938 PMC3271177

[9] Barouch DH, Alter G, Broge T, Linde C, Ackerman ME, Brown EP, Borducchi EN, Smith KM, Nkolola JP, Liu J, Shields J, Parenteau L, Whitney JB, Abbink P, Ng'ang'a DM, Seaman MS, Lavine CL, Perry JR, Li W, Colantonio AD, Lewis MG, Chen B, Wenschuh H, Reimer U, Piatak M, Lifson JD, Handley SA, Virgin HW, Koutsoukos M, Lorin C, Voss G, Weijtens M, Pau MG, Schuitemaker H 2015 Protective efficacy of adenovirus/protein vaccines against SIV challenges in rhesus monkeys. Science 349:320–324. doi:10.1126/science.aab3886. 26138104 PMC4653134

[10] Lai L, Kwa SF, Kozlowski PA, Montefiori DC, Nolen TL, Hudgens MG, Johnson WE, Ferrari G, Hirsch VM, Felber BK, Pavlakis GN, Earl PL, Moss B, Amara RR, Robinson HL 2012 SIVmac239 MVA vaccine with and without a DNA prime, similar prevention of infection by a repeated dose SIVsmE660 challenge despite different immune responses. Vaccine 30:1737–1745. doi:10.1016/j.vaccine.2011.12.026. 22178526 PMC3278564

[11] Roederer M, Keele BF, Schmidt SD, Mason RD, Welles HC, Fischer W, Labranche C, Foulds KE, Louder MK, Yang ZY, Todd JP, Buzby AP, Mach LV, Shen L, Seaton KE, Ward BM, Bailer RT, Gottardo R, Gu W, Ferrari G, Alam SM, Denny TN, Montefiori DC, Tomaras GD, Korber BT, Nason MC, Seder RA, Koup RA, Letvin NL, Rao SS, Nabel GJ, Mascola JR 2014 Immunological and virological mechanisms of vaccine-mediated protection against SIV and HIV. Nature 505:502–508. doi:10.1038/nature12893. 24352234 PMC3946913

[12] Rerks-Ngarm S, Pitisuttithum P, Nitayaphan S, Kaewkungwal J, Chiu J, Paris R, Premsri N, Namwat C, de Souza M, Adams E, Benenson M, Gurunathan S, Tartaglia J, McNeil JG, Francis DP, Stablein D, Birx DL, Chunsuttiwat S, Khamboonruang C, Thongcharoen P, Robb ML, Michael NL, Kunasol P, Kim JH, MOPH-TAVEG Investigators. 2009 Vaccination with ALVAC and AIDSVAX to prevent HIV-1 infection in Thailand. N Engl J Med 361:2209–2220. doi:10.1056/NEJMoa0908492. 19843557

[13] Hansen SG, Sacha JB, Hughes CM, Ford JC, Burwitz BJ, Scholz I, Gilbride RM, Lewis MS, Gilliam AN, Ventura AB, Malouli D, Xu GW, Richards R, Whizin N, Reed JS, Hammond KB, Fischer M, Turner JM, Legasse AW, Axthelm MK, Edlefsen PT, Nelson JA, Lifson JD, Fruh K, Picker LJ 2013 Cytomegalovirus vectors violate CD8(+) T cell epitope recognition paradigms. Science 340:1237874–1237957. doi:10.1126/science.1237874. 23704576 PMC3816976

[14] Hansen SG, Vieville C, Whizin N, Coyne-Johnson L, Siess DC, Drummond DD, Legasse AW, Axthelm MK, Oswald K, Trubey CM, Piatak M, Lifson JD, Nelson JA, Jarvis MA, Picker LJ 2009 Effector memory T cell responses are associated with protection of rhesus monkeys from mucosal simian immunodeficiency virus challenge. Nat Med 15:293–299. doi:10.1038/nm.1935. 19219024 PMC2720091

[15] Hansen SG, Wu HL, Burwitz BJ, Hughes CM, Hammond KB, Ventura AB, Reed JS, Gilbride RM, Ainslie E, Morrow DW, Ford JC, Selseth AN, Pathak R, Malouli D, Legasse AW, Axthelm MK, Nelson JA, Gillespie GM, Walters LC, Brackenridge S, Sharpe HR, Lopez CA, Fruh K, Korber BT, McMichael AJ, Gnanakaran S, Sacha JB, Picker LJ 2016 Broadly targeted CD8(+) T cell responses restricted by major histocompatibility complex E. Science 351:714–720. doi:10.1126/science.aac9475. 26797147 PMC4769032

[16] Hansen SG, Ford JC, Lewis MS, Ventura AB, Hughes CM, Coyne-Johnson L, Whizin N, Oswald K, Shoemaker R, Swanson T, Legasse AW, Chiuchiolo MJ, Parks CL, Axthelm MK, Nelson JA, Jarvis MA, Piatak M Jr, Lifson JD, Picker LJ 2011 Profound early control of highly pathogenic SIV by an effector memory T-cell vaccine. Nature 473:523–527. doi:10.1038/nature10003. 21562493 PMC3102768

[17] Murray SE, Nesterenko PA, Vanarsdall AL, Munks MW, Smart SM, Veziroglu EM, Sagario LC, Lee R, Claas FHJ, Doxiadis IIN, McVoy MA, Adler SP, Hill AB 2017 Fibroblast-adapted human CMV vaccines elicit predominantly conventional CD8 T cell responses in humans. J Exp Med 214:1889–1899. doi:10.1084/jem.20161988. 28566275 PMC5502433

[18] Lawler SH, Sussman RW, Taylor LL 1995 Mitochondrial DNA of the Mauritian macaques (Macaca fascicularis): an example of the founder effect. Am J Phys Anthropol 96:133–141. doi:10.1002/ajpa.1330960203. 7755104

[19] Mee ET, Badhan A, Karl JA, Wiseman RW, Cutler K, Knapp LA, Almond N, O’Connor DH, Rose NJ 2009 MHC haplotype frequencies in a UK breeding colony of Mauritian cynomolgus macaques mirror those found in a distinct population from the same geographic origin. J Med Primatol 38:1–14. doi:10.1111/j.1600-0684.2008.00299.x. PMC4509677 19018947

[20] Rodriguez F, Zhang J, Whitton JL 1997 DNA immunization: ubiquitination of a viral protein enhances cytotoxic T-lymphocyte induction and antiviral protection but abrogates antibody induction. J Virol 71:8497–8503. https://jvi.asm.org/content/71/11/8497. 9343207 10.1128/jvi.71.11.8497-8503.1997 PMC192313

[21] Tobery TW, Siliciano RF 1997 Targeting of HIV-1 antigens for rapid intracellular degradation enhances cytotoxic T lymphocyte (CTL) recognition and the induction of de novo CTL responses in vivo after immunization. J Exp Med 185:909–920. doi:10.1084/jem.185.5.909. 9120397 PMC2196169

[22] Townsend A, Bastin J, Gould K, Brownlee G, Andrew M, Coupar B, Boyle D, Chan S, Smith G 1988 Defective presentation to class-I-restricted cytotoxic T lymphocytes in vaccinia-infected cells is overcome by enhanced degradation of antigen. J Exp Med 168:1211–1224. doi:10.1084/jem.168.4.1211. 2459295 PMC2189091

[23] Benlahrech A, Meiser A, Herath S, Papagatsias T, Athanasopoulos T, Li F, Self S, Bachy V, Hervouet C, Logan K, Klavinskis L, Dickson G, Patterson S 2012 Fragmentation of SIV-gag vaccine induces broader T cell responses. PLoS One 7:e48038. doi:10.1371/journal.pone.0048038. 23118924 PMC3485275

[24] Kedl RM, Rees KA, Hildeman DA, Schaefer B, Mitchell T, Kappler J, Marrack P 2000 T cells compete for access to antigen-bearing antigen-presenting cells. J Exp Med 192:1105–1113. doi:10.1084/jem.192.8.1105. 11034600 PMC2195874

[25] Liu Y, Li FS, Liu Y, Hong KX, Meng X, Chen JP, Zhang Z, Huo Z, Sun MS, Self SG, Shao YM 2011 HIV fragment gag vaccine induces broader T cell response in mice. Vaccine 29:2582–2589. doi:10.1016/j.vaccine.2011.01.049. 21292005

[26] Singh RAK, Barry MA 2004 Repertoire and immunofocusing of CD8 T cell responses generated by HIV-1 gag-pol and expression library immunization vaccines. J Immunol 173:4387–4393. doi:10.4049/jimmunol.173.7.4387. 15383568

[27] Casimiro DR, Wang F, Schleif WA, Liang X, Zhang Z-Q, Tobery TW, Davies M-E, McDermott AB, O'Connor DH, Fridman A, Bagchi A, Tussey LG, Bett AJ, Finnefrock AC, Fu TM, Tang A, Wilson KA, Chen M, Perry HC, Heidecker GJ, Freed DC, Carella A, Punt KS, Sykes KJ, Huang L, Ausensi VI, Bachinsky M, Sadasivan-Nair U, Watkins DI, Emini EA, Shiver JW 2005 Attenuation of simian immunodeficiency virus SIVmac239 infection by prophylactic immunization with DNA and recombinant adenoviral vaccine vectors expressing Gag. J Virol 79:15547–15555. doi:10.1128/JVI.79.24.15547-15555.2005. 16306625 PMC1315991

[28] Cicala C, Martinelli E, McNally JP, Goode DJ, Gopaul R, Hiatt J, Jelicic K, Kottilil S, Macleod K, O'Shea A, Patel N, Van Ryk D, Wei D, Pascuccio M, Yi L, McKinnon L, Izulla P, Kimani J, Kaul R, Fauci AS, Arthos J 2009 The integrin alpha4beta7 forms a complex with cell-surface CD4 and defines a T-cell subset that is highly susceptible to infection by HIV-1. Proc Natl Acad Sci U S A 106:20877–20882. doi:10.1073/pnas.0911796106. 19933330 PMC2780317

[29] Herath S, Benlahrech A, Papagatsias T, Athanasopoulos T, Bouzeboudjen Z, Hervouet C, Klavinskis L, Meiser A, Kelleher P, Dickson G, Patterson S 2014 Fusion of ubiquitin to HIV Gag impairs human monocyte-derived dendritic cell maturation and reduces ability to induce Gag T cell responses. PLoS One 9:e88327. doi:10.1371/journal.pone.0088327. 24505475 PMC3914991

[30] Vidalin O, Tanaka E, Spengler U, Trepo C, Inchauspe G 1999 Targeting of hepatitis C virus core protein for MHC I or MHC II presentation does not enhance induction of immune responses to DNA vaccination. DNA Cell Biol 18:611–621. doi:10.1089/104454999315024. 10463057

[31] Budde ML, Greene JM, Chin EN, Ericsen AJ, Scarlotta M, Cain BT, Pham NH, Becker EA, Harris M, Weinfurter JT, O'Connor SL, Piatak M, Lifson JD, Gostick E, Price DA, Friedrich TC, O'Connor DH 2012 Specific CD8^+^ T cell responses correlate with control of simian immunodeficiency virus replication in Mauritian cynomolgus macaques. J Virol 86:7596–7604. doi:10.1128/JVI.00716-12. 22573864 PMC3416303

[32] Sutton MS, Ellis-Connell A, Moriarty RV, Balgeman AJ, Gellerup D, Barry G, Weiler AM, Friedrich TC, O'Connor SL 2018 Acute-phase CD4(+) T cell responses targeting invariant viral regions are associated with control of live attenuated simian immunodeficiency virus. J Virol 92:e00830-18. doi:10.1128/JVI.00830-18. 30111562 PMC6189504

[33] Yovandich JL, Chertova EN, Kane BP, Gagliardi TD, Bess JW Jr, Sowder RC II, Henderson LE, Gorelick RJ 2001 Alteration of zinc-binding residues of simian immunodeficiency virus p8(NC) results in subtle differences in gag processing and virion maturation associated with degradative loss of mutant NC. J Virol 75:115–124. doi:10.1128/JVI.75.1.115-124.2001. 11119580 PMC113904

[34] Reynolds MR, Weiler AM, Piaskowski SM, Piatak M, Robertson HT, Allison DB, Bett AJ, Casimiro DR, Shiver JW, Wilson NA, Lifson JD, Koff WC, Watkins DI 2012 A trivalent recombinant Ad5 gag/pol/nef vaccine fails to protect rhesus macaques from infection or control virus replication after a limiting-dose heterologous SIV challenge. Vaccine 30:4465–4475. doi:10.1016/j.vaccine.2012.04.082. 22569124 PMC3372643

[35] Conti P, Barbacane RC, Feliciani C, Reale M 2001 Expression and secretion of RANTES by human peripheral blood CD4+ cells are dependent on the presence of monocytes. Ann Clin Lab Sci 31:75–84. http://www.annclinlabsci.org/content/31/1/75.full?sid=2e224f91-c9a1-435f-ad22-e7007401b135. 11314865

[36] Lewis DJ, Wang Y, Huo Z, Giemza R, Babaahmady K, Rahman D, Shattock RJ, Singh M, Lehner T 2014 Effect of vaginal immunization with HIVgp140 and HSP70 on HIV-1 replication and innate and T cell adaptive immunity in women. J Virol 88:11648–11657. doi:10.1128/JVI.01621-14. 25008917 PMC4178755

[37] Chicz RM, Urban RG, Gorga JC, Vignali DA, Lane WS, Strominger JL 1993 Specificity and promiscuity among naturally processed peptides bound to HLA-DR alleles. J Exp Med 178:27–47. doi:10.1084/jem.178.1.27. 8315383 PMC2191090

[38] Mackewicz CE, Blackbourn DJ, Levy JA 1995 CD8+ T cells suppress human immunodeficiency virus replication by inhibiting viral transcription. Proc Natl Acad Sci U S A 92:2308–2312. doi:10.1073/pnas.92.6.2308. 7534418 PMC42473

[39] Reimann KA, Parker RA, Seaman MS, Beaudry K, Beddall M, Peterson L, Williams KC, Veazey RS, Montefiori DC, Mascola JR, Nabel GJ, Letvin NL 2005 Pathogenicity of simian-human immunodeficiency virus SHIV-89.6P and SIVmac is attenuated in cynomolgus macaques and associated with early T-lymphocyte responses. J Virol 79:8878–8885. doi:10.1128/JVI.79.14.8878-8885.2005. 15994781 PMC1168747

[40] Ferguson D, Wade-Evans A, Elsley W, Sangster R, Silvera P, MacManus S, Davis G, Corcoran T, Berry N, Brown S, Jenkins A, Cowie J, Sethi M, Hull R, Stebbings R, Lines J, Norley S, Stott EJ, Almond N 2007 Preparation and characterization of new challenge stocks of SIVmac32H J5 following rapid serial passage of virus in vivo. J Med Primatol 36:131–142. doi:10.1111/j.1600-0684.2007.00224.x. 17517087

[41] O’Connor SL, Lhost JJ, Becker EA, Detmer AM, Johnson RC, Macnair CE, Wiseman RW, Karl JA, Greene JM, Burwitz BJ, Bimber BN, Lank SM, Tuscher JJ, Mee ET, Rose NJ, Desrosiers RC, Hughes AL, Friedrich TC, Carrington M, O’Connor DH 2010 MHC heterozygote advantage in simian immunodeficiency virus-infected Mauritian cynomolgus macaques. Sci Transl Med 2:22ra18. doi:10.1126/scitranslmed.3000524. PMC2865159 20375000

[42] Tenbusch M, Ignatius R, Temchura V, Nabi G, Tippler B, Stewart-Jones G, Salazar AM, Sauermann U, Stahl-Hennig C, Uberla K 2012 Risk of immunodeficiency virus infection may increase with vaccine-induced immune response. J Virol 86:10533–10539. doi:10.1128/JVI.00796-12. 22811518 PMC3457298

[43] Sauermann U, Radaelli A, Stolte-Leeb N, Raue K, Bissa M, Zanotto C, Krawczak M, Tenbusch M, Uberla K, Keele BF, De Giuli Morghen C, Sopper S, Stahl-Hennig C 2017 Vector order determines protection against pathogenic simian immunodeficiency virus infection in a triple component vaccine by balancing CD4(+) and CD8(+) T-cell responses. J Virol 17:e01120-17. doi:10.1128/JVI.01120-17. PMC5686736 28904195

[44] Berry N, Stebbings R, Ferguson D, Ham C, Alden J, Brown S, Jenkins A, Lines J, Duffy L, Davis L, Elsley W, Page M, Hull R, Stott J, Almond N 2008 Resistance to superinfection by a vigorously replicating, uncloned stock of simian immunodeficiency virus (SIVmac251) stimulates replication of a live attenuated virus vaccine (SIVmacC8). J Gen Virol 89:2240–2251. doi:10.1099/vir.0.2008/001693-0. 18753233

[45] Bolger AM, Lohse M, Usadel B 2014 Trimmomatic: a flexible trimmer for Illumina sequence data. Bioinformatics 30:2114–2120. doi:10.1093/bioinformatics/btu170. 24695404 PMC4103590

[46] Li H, Durbin R 2009 Fast and accurate short read alignment with Burrows-Wheeler transform. Bioinformatics 25:1754–1760. doi:10.1093/bioinformatics/btp324. 19451168 PMC2705234

